# Our Delay

**Published:** 1840

**Authors:** 


### WESTERN JOURNAL.

No's. 2, 3, 4 & 5.

LOUISVILLE, MAY 31, 1840.

---

### OUR DELAY.

*Dr. Franklin*, or some other sage, has said, that he who is good at an *apology* is seldom good for any thing else. That we may dodge the point of this sarcasm, we shall take care not to make the best apology in the world, for the suspension of our enterprise from January to June. Such as it is—here it comes. When our first number was *in transitu* from the printing to the post-office, some *premonitory symptoms of disease* in the LOUISVILLE MEDICAL INSTITUTE, began to show themselves, and soon became so threatening, that our publishers, with the prudence of sound business men, were inclined from the connexion between the *Journal* and the *Institute*, to lie by, till they should see whether the *forming disease*, was likely to inflict any serious *organic lesion* on the latter. The *morbid action* took its course, the *vis conservatrix* awoke, a *crisis* occurred, *convalescence* followed, and *sound health* is restored. The first fruits of this recovery are four numbers of the Journal at one birth, with the prospect of a regular monthly delivery, for an indefinite time hereafter. D.

